# Assessment of the Drone Arm’s Plastic–Metal Joint Mechanical Resistance Following Natural and Artificial Aging of the 3D-Printed Plastic Component

**DOI:** 10.3390/ma18112591

**Published:** 2025-06-01

**Authors:** Miloš R. Vasić, Snežana Vučetić, Vesna Miljić, Miloš Vorkapić, Anja Terzić, Mladen Ćosić, Danica M. Bajić

**Affiliations:** 1 Institute for Testing of Materials, Bulevar Vojvode Mišića 43, 11000 Belgrade, Serbia; milos.vasic@institutims.rs (M.R.V.); mladen.cosic@institutims.rs (M.Ć.); 2 Faculty of Technology, University of Novi Sad, Bulevar cara Lazara 1, 21000 Novi Sad, Serbia; snezanap@uns.ac.rs (S.V.); vesna.kizic@uns.ac.rs (V.M.); 3 Institute of Chemistry, Technology and Metallurgy, National Institute of the Republic of Serbia, University of Belgrade, Njegoševa 12, 11000 Belgrade, Serbia; worcky@nanosys.ihtm.bg.ac.rs; 4 Military Technical Institute, Ratka Resanovića 1, 11030 Belgrade, Serbia; danica.bajic@mod.gov.rs

**Keywords:** additive manufacturing, mechanical properties, heat staking process, numerical modeling, PLA

## Abstract

As drone technologies advance, there is an increasing need for structural components that are lightweight, durable, and easily replaceable. Additive manufacturing (AM) with PLA offers a cost-effective solution to improve mechanical performance, especially when enhanced with embedded metal inserts. However, the long-term durability of PLA–metal joints under environmental aging conditions remains underexplored. This study evaluates the mechanical integrity of 3D-printed PLA drone arms produced with reduced infill density with embedded brass inserts. To replicate realistic service conditions, the samples underwent natural aging and five artificial aging protocols involving thermal cycling, humidity, UV/IR exposure, and freeze–thaw cycles. Mechanical performance was assessed through pull-out and tensile strength testing, supported by FTIR spectroscopy, colorimetric, wettability analysis, and finite element modeling. Notably, to our knowledge, wettability analysis has not previously been applied to this type of material, and metal–plastic contact zones have not been tested under such comprehensive aging protocols. Results showed a 70% reduction in pull-out strength under harsh conditions, though the joints remained functional. Numerical modeling confirmed that stress concentration begins on the inner side of the arm. With optimal print settings, the arm can support a 2.31 kg payload (20% confidence), while the metal inserts withstand up to 17.9 kg.

## 1. Introduction

Additive manufacturing (AM) is a well-accepted and widely utilized method for producing components, prototypes, and parts, as well as customized tools and accessories through a layer-by-layer construction process. AM is constantly evolving to expand and find its place in as many possible different industrial sectors, including AEC (Architecture-Engineering-Construction) [1]. One of the most prominent advantages of this technology is the quick manufacture of geometrically difficult pieces, which is even more applicable if serial production is not necessary [2].

Metal threads are utilized for joining materials in a variety of industries, including metal structures, shipbuilding, automobiles, electronics, aircraft, and defense. Its application is widening, especially when rapid construction and disassembly of components are required [3]. There is an absence of detailed studies which analyze the quality of AM-produced plastic parts in the metal–plastic joints. Metal–plastic joints are essential because their quality influences the final product’s performances. The construction industry has simply embraced metal and plastic joints during the assembly process, which demands rapid connection of smaller pieces [4]. The maximal pull-out force is a widely accepted criterion for quantifying the quality of the metal–plastic joints. During test, the force required to pull the threaded insert out of the plastic is continually measured. This method is still not standardized, even though the force can be measured on standard pull-out or tensile strength test devices. Thus, researchers are not always using the same preset parameters (e.g., load speed, sample dimensions, etc.) in their investigations [5].

Several methods (insert molding, thermal, cold, and ultrasonic insertion) are available for joining thermoplastic material with metal inserts [6,7]. Amancio et al. [8] analyzed three techniques for joining polymers and their composites: mechanical fastening (screws or rivets), adhesive bonding, and welding (melting the materials to form a joint). Each of these techniques has its advantages and disadvantages. Namely, mechanical joining has certain difficulties due to the different physical and chemical properties of metals and polymers (thermal expansion, surface energy, and mechanical strength). Additionally, the characteristics of the used materials have a huge impact on the durability and design of the final products. Metal inserts (or threatened inserts) offer better qualities than printed parts in which they are embedded, particularly regarding the stress–strain relationships.

Heat staking is a commonly used technique for joining metal and printed plastic. The main advantage of this method is that the joining process does not require drilling that additionally affects the mechanical properties of the plastic parts obtained by AM [3]. During embedding, the softened plastic adheres to the outer surface of the insert. This procedure can be achieved using different processes [9]. The strength of the joint is greatly influenced by the joining technology, the geometry of the insert, and preset printing parameters, for example, the specified number of top and bottom layers and wall line contours [10]. The strongest connections are made by directly sinking metal threaded inserts into the plastic [5]. Fürst et al. [11] presented a procedure in which heat input is delivered via outer thread flanks during application. This method can increase the overall strength of the joint compared to the standard heat staking procedure. Miklavec et al. [12] used different forms of metal inserts to improve the mechanical characteristics of the hybrid joint. Results have confirmed that the design of metal inserts, especially its outside surface, is an essential parameter for ensuring better polymer bonding to the metal, which consequently leads to the increase of its strength and corrosion resistance. Furthermore, the folds on the outside of the metal inserts have a direct impact on pull-out resistance and torque [13]. An investigation into the possibilities of enhancing the stiffness and mechanical strength of the composite carbon fiber reinforced plastic is another example of how the geometry and form of the metal insert impact the joint [14]. Additional bed patterns were added to the metal sheet of the insert. This technology significantly increased the performance of composite products, particularly their load-bearing capability. Thereby, composite components can be robustly linked with other materials. The influence of printing parameters on the quality of the joint between the insert and plastic was reported in the study by Kastner et al. [15]. It was found that infill density significantly affects the joint strength. A direct relation between infill density and the pulling force was observed. Optimal printing preset was also reported. Recommended infill density, wall thickness, layer height, and print temperature were set to 70%, 1.2 mm, 0.2 mm, and 225 °C, respectively. When it comes to sandwich panels, the performance of inserts subjected to pulling forces was also investigated. For example, Seeman et al. [16] employed numerical models to predict pull-out strength, whereas Stefan et al. [17] experimentally investigated the mechanical properties of panels with inserted metal joints under various loading conditions.

Degradation is commonly seen as the deleterious distortion of the material: surface appearance (color change), chemical structure, or physical properties. In the case of a polymer, degradation occurs as a result of macromolecule chemical cleavage. Various mechanisms (photo-oxidative, thermo-oxidative, ozone-induced, mechanochemical, hydrolytic, freeze–thaw, and biodegradation) can act separately or simultaneously and cause polymer chain scission. Polymer aging is commonly seen as the gradual degradation of material over time caused by environmental factors such as heat, moisture, oxygen exposure, light (UV radiation), or mechanical stress [18,19,20].

Accelerated aging refers to any artificial procedure that increases one or more variables affecting the material’s natural decay. The primary goal is to simulate the long-term effects of environmental conditions in a shorter time frame. Orellana-Barrasa et al. [21] aged PLA in a temperature range from 20 to 80 °C. It was found that PLA can be safely aged without degrading at 39 °C. Vorkapić et al. [22] studied the impact of temperature aging at 57 °C on the tensile properties of PLA printed samples. It was found that with aging, the mechanical properties decreased. This is more pronounced for samples printed with higher layer heights. Products with 0/90 [°] orientation were mostly brittle, while products printed using −45/45 [°] orientation withstood the highest deformations before failure. The impact of UV radiation on PLA samples can be found, for example, in Refs. [23,24,25]. Refs. [25,26] described PLA weathering tests utilizing the methodology that included two (humidity and temperature) and three (humidity, temperature, and UV) aging agents. The main finding was that mechanical properties were lower for samples aged at maximal relative humidity content.

Current methods for assessing the long-term performance of polymers are limited and should be interpreted with caution, particularly when multiple degradation factors are present. A key issue is that most weathering standards focus on individual degrading agents, rather than their combined effects. Moreover, there is a notable absence of ISO or ASTM aging standards that simulate cyclic laboratory conditions incorporating all relevant degradation mechanisms such as thermal, humidity, and weathering exposure [27].

The state-of-the-art focuses mostly on the static mechanical performance of PLA–metal joints, with little information available on their behavior after environmental degradation, notably in the context of drone or structural applications. Existing studies have largely employed single-factor artificial aging protocols, such as exposure to UV radiation or elevated temperatures or humidity. This study addresses this evident gap in knowledge by introducing and applying multi-factor aging simulations that more accurately reflect real-world environmental conditions. Furthermore, the structural application of low-infill-density 3D-printed components was overlooked due to widely held beliefs about their mechanical limitations. The current study challenges this perception and investigates if satisfactory functional performance can be achieved even after prolonged aging. Furthermore, a lack of standardized procedures for pull-out testing of metal threads embedded in plastic is identified. To solve this, a customized adapter was designed, allowing for a consistent and systematic testing method.

Since plastic materials degrade over time, the primary goal of this study was to verify that the PLA–metal joint remains functional after aging. This study is among the first to systematically evaluate the mechanical integrity of PLA–brass heat staked joints subjected to both natural and five novel artificial aging protocols. These protocols used a combination of heat cycling, humidity, UV, and IR exposure, as well as severe and extreme freeze–thaw conditions, to simulate real-world environmental degradation. A comprehensive, multi-modal assessment approach that combines pull-out testing, FTIR spectroscopy, colorimetric analysis, wettability measurements, and finite element method (FEM) simulations to evaluate degradation and performance is proposed. Additionally, a novel modular drone arm design incorporating threaded brass inserts was developed to enhance field repairability and durability. The main objective is to assess whether adequate structural integrity of the drone arm, printed with unrecommended settings, namely, a reduced infill density of 35%, could still be achieved and preserved after aging, thereby supporting more accessible and cost-effective AM.

## 2. Materials and Methods

### 2.1. Drone and Drone Arm Design

**Figure 1:**
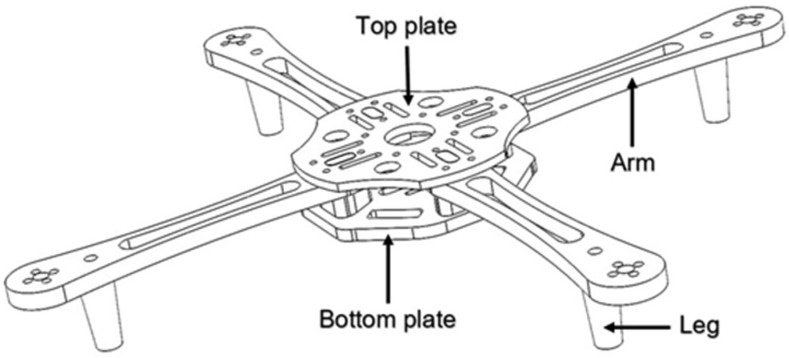
Scheme of the experimental drone frame design.

The drone arm design is important for both mechanical and dynamic loading. The arm cross-section is crucial from the aspect of load-bearing capacity (i.e., strength) and satisfying aerodynamic properties [28]. Drone arms usually have a circular or rectangular cross-section, but can also be triangular or T-shaped. The rectangular cross-section is the strongest and provides the required mechanical performance, while the circular cross-section is the most aerodynamically efficient [29,30,31]. The drone arm designed in this experiment is set to be tested on a drone whose purpose will be identified according to the defined payload [30]. An analysis of attaching the arm drone with other frame elements was conducted in this paper based on the guidelines and suggestions for the design of an unmanned aerial vehicle (UAV) [29,32,33,34,35,36]. As illustrated in Figure 1, threaded inserts are pressed into the drone arms using heat staking technology. The technique of arms connecting to the top and bottom plates is done by pressing the protrusions on the arms into specially designed holes on the plate.

**Figure 2:**
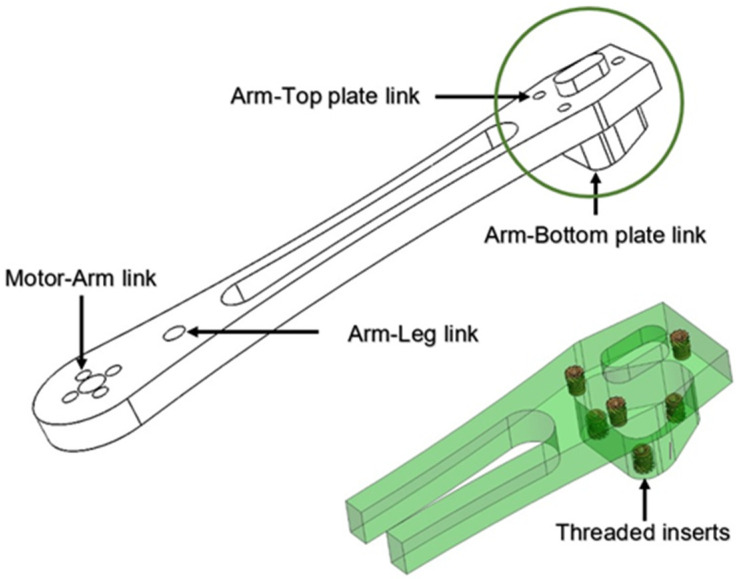
Scheme of the threaded inserts placement in the designed drone arm.

The links between the plates, the arms, and the legs are solid and compact. Although similar modular solutions were described in the literature [30,37,38,39,40], the design of the central part of the drone frame is new. An inner cylinder between the upper and lower plates can accommodate four, six, or eight arms. The connection of the cylinder to the arms is achieved by threaded inserts. In the case of minor impacts or collisions, the connection of the shoulder to the plates allows the impact to be absorbed and other components to be protected. The position of the brass inserts in the drone arm is illustrated in Figure 2. The connection via threaded inserts enables the arm to be replaced quickly after damage. Modularity and the connection of the cylinder to the arms achieved by threaded inserts can help in expanding the drone life-cycle and reducing the repair costs.

### 2.2. Employed Materials and Methodology

A commercial white PLA thermoplastic filament with a diameter of 1.75 mm was used. According to Table 1 (for complete Creality data sheet see Ref. [41]) the mechanical properties (tensile, bending, and impact strength) of the PLA thermoplastic comply with the preset project requirements for the drone arm’s intended use. This material is derived from renewable sources and belongs to the group of biodegradable materials [42,43].

Before printing, the 3D CAD technical drawing is converted to STL format, which is then transformed to G-code because it is the only standardized native file that printing device software can recognize and use. All samples were printed on a Creality Ender-6 device using an extruder with a nozzle diameter of 0.4 mm. The preset printing parameters are given in Table 2. The other parameters were as follows: print temperature 210 °C, bed temperature 60 °C, infill speed 60 mm/s, infill density 35%, raster line 0.3, and infill line direction −45°/+45°. The infill pattern zig-zag and travel speed 80 mm/s were constant.

**Figure 3:**
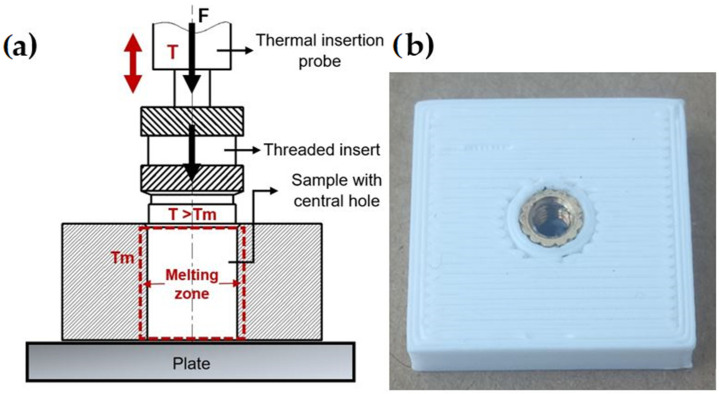
Heat staking procedure: (**a**) procedure’ schematic view; (**b**) final appearance of the metal–plastic output.

The metal brass inserts [44] were embedded into the samples using the heat staking procedure (illustrated in Figure 3a). The metal insert was initially placed vertically on the surface of the sample. A soldering iron which was previously heated to the temperature of 230 °C was placed inside the metal insert, and the insertion process was carried out. Once the insert was aligned with the sample, the soldering iron was removed. Special care was taken to ensure that during insertion a minimal force was applied, as recommended in Refs. [7,45,46]. The soldering iron was mounted on a custom-designed stand that enabled precise vertical adjustment. This configuration ensured that the iron could be consistently positioned at the same height during insertion. By standardizing the contact position, the setup significantly improved the repeatability of the process and minimized errors associated with excessive manual pressure during soldering. During this process, the heat spread through the brass was used to liquefy the plastic within a close narrow zone commonly called melting (Figure 3b). The plastic flows around the brass inserts are registered in this zone.

According to the literature [40], increasing the extruder temperature to the range of 190–230 °C during printing contributes to a desirable reduction in the weight of printed PLA products, while causing only a slight decrease in mechanical properties. This outcome is particularly valuable for the development of lightweight applications.

The soldering iron probe should not overheat the insert, as this would cause the thermoplastic material to burn and the hole to expand, resulting in a loose connection [47]. Furthermore, the dimension of the central hole has a direct impact on the adhesion bond between the metal insert and the thermoplastic polymer. It is vital to carefully determine the diameter of the hole. If it exceeds the outer diameter of the metal insert, a poor connection will be formed during the heat staking procedure. (Figure 3b). Conversely, increasing the impact force causes more plastic to be extruded. The hole ensures a secure connection only once the metal insert is completely lodged in the plastic [48,49,50].

**Figure 4:**
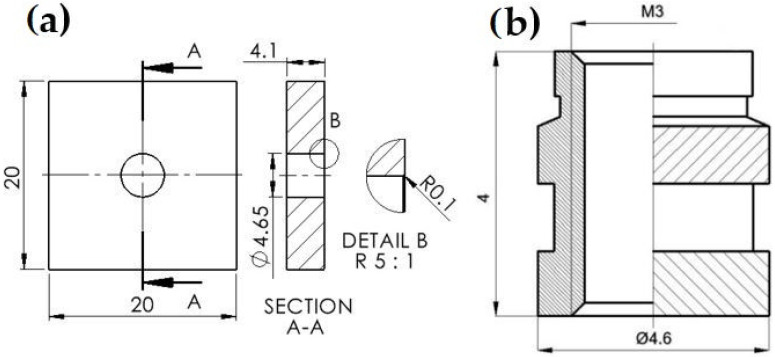
PLA sample: (**a**) the scheme of the sample; (**b**) brass element dimensions.

During printing, dimensional deviations can occur as a consequence of material shrinkage, layer adhesion, or inadequate printer calibration. In this case, inadequate printer calibration can be excluded, since regular maintenance (checking the filament storage, printer alignment, and nozzle cleaning) was applied. The fact that no warping impact was identified on printed samples suggests that the second-factor layer adhesion is satisfactory. Minor dimensional differences are mostly caused by the PLA filament contracting while cooling; therefore, the central hole was larger than the brass insert, as shown in Figure 4a.

**Figure 5:**
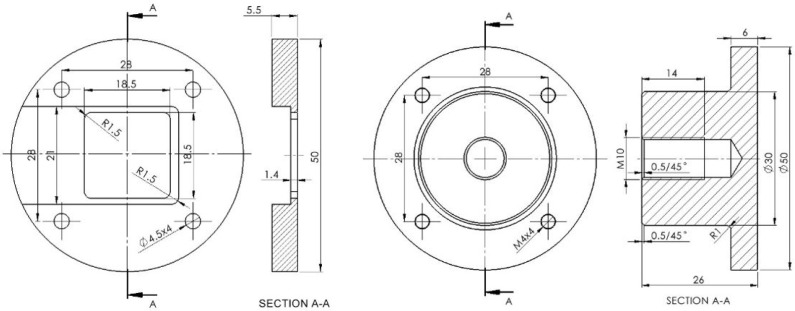
Schematic view of the adapter tool.

Samples with inserted brass (Figure 4b) were naturally and artificially aged. After aging, the mechanical quality of the joint between the plastic and metal inserts was evaluated. The pull-out force was determined. Measurements were conducted on the laboratory CONTROLS pull-off tester and the Shimadzu compact table-top universal tensile tester. Both devices have a capacity of 5 kN. A specialized adaptor tool was built for the experiment. It was utilized as a sample holder for the tensile strength tests. The tool is composed of aluminum and created using traditional machining techniques (lathe, milling machine). It is made of two components, as illustrated in Figure 5.

**Figure 6:**
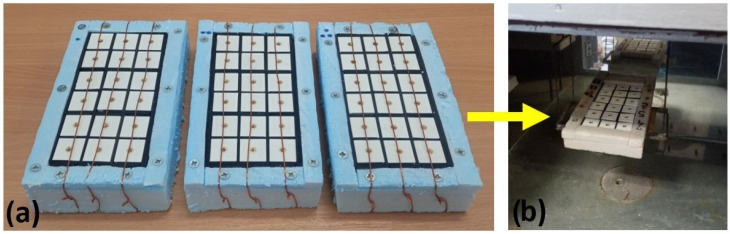
Illustration of test panels: (**a**) disposition of the samples; (**b**) the artificial aging procedure.

The metal–plastic samples were initially analyzed two hours after brass implantation. The test designation code of the initial sample is SP. The natural aging procedure (NP) means that samples were examined after three months and after one year of curing at ambient temperature (approximately 20 °C). Several test panels were constructed, as illustrated in Figure 6a. The first panel row was created by combining three samples from the first printing combination. The next row represents the second printing combination. This pattern was repeated up to the sixth panel row. Each sample was isolated from all sides except the upper side using a Styrofoam and a rubber joint.

The initial artificial aging protocol (AP-I) consisted of 22 cycles (Figure 6b). Samples were aged during 44 days in total in the Binder KBWF 240 climate chamber equipped with 2 illumination cassettes with IR and UV light. One cycle lasted for two days. During the first day, the winter weathering conditions were simulated. Temperature and relative humidity were 5 °C and 80%. On the second day, the summer weathering conditions were simulated. Temperature and relative humidity were 40 °C and 25%. The panel was removed from the chamber after 14, 29, 36, and 44 days. FTIR analysis, colorimetry, and wettability tests were conducted on the 14th, 29th, 36th, and 44th day. After conducting the analyses, the test panels were assembled again and returned to the climate chamber. The contact angle measurement was performed using the Surface Energy Evaluation System, Advex Instruments, Brno, Czech Republic, with demineralized water as the measuring fluid. FTIR analysis was conducted using the Alpha (BRUKER Optics, Ettlingen, Germany) FTIR instrument with a DRIFT (Diffuse Reflectance) attachment for non-contact measurements, in the wavenumber range of 400 to 4000 cm^−1^ and a resolution of 4 cm^−1^. The FTIR spectrum for each measurement position is an averaged spectrum of 24 scans. The obtained FTIR spectra were then analyzed using the OPUS software (version 1.12.16.1). The spectrograph software version 1.12.16.1 was used for plotting the FTIR raw data and finding peak values [50].

The FTIR spectra were analyzed using absolute absorbance value and degradation index (DI). The choice depends on the specific focus of the analysis. Absolute absorbance is particularly useful for spatial or compositional comparisons, as it reflects the direct presence and accumulation of specific chemical groups within different regions of the sample. This approach is well-suited for identifying localized chemical variations, such as those occurring at interfaces or between exposed and protected surfaces. In contrast, the degradation index is more appropriate for time-based tracking or assessing the progression of degradation. By comparing relative changes in functional group absorbance over time, DI provides a normalized measure that highlights increases or decreases in specific bonds or chemical structures, thereby offering insight into the rate and extent of chemical degradation. Together, both methods complement each other and can provide a comprehensive understanding of material changes under varying conditions.

The second artificial aging protocol (AP-II) included 28 days of aging in a climate chamber without an IR and a UV lamp. A test panel was initially kept in the air for 8 h at 80% relative humidity, 40 °C temperature, and 1 m/s velocity. Afterward, the panel was taken out and left in the desiccator for the following 16 h at 25 °C. One cycle lasted for 24 h. Sample mass change during the initial 8 h of each cycle was recorded. Temperature, humidity, and velocity were regulated inside the chamber with the accuracy of ±0.2 °C, 0.2%, and 0.1 m/s, respectively. Mass was recorded with an accuracy of 0.01 g.

The following three artificial aging protocols (AP-III, AP-IV, and AP-V) monitored the long-term durability of materials through freeze–thaw resistance tests conducted under severe and extreme conditions. Two durability tests were performed under severe conditions. The first test (AP-III) was based on the EN 772-22:2018 standard. The test panel was placed in a freeze–thaw slab tester, where each cycle consisted of a freezing and thawing phase. The air temperature, measured 40 ± 10 mm from the exposed surface, was gradually reduced from 20 °C to −15 °C, over 30 min. The panel was then kept at −15 °C for 100 min. Warm air was induced to facilitate the thawing. This phase was limited to 20 min. Upon completion of 100 freeze–thaw cycles, the panel was disassembled, and each sample was carefully examined for defects such as surface cracks, through-cracks, scaling, chipping, or peeling. During the second test (AP-IV), the panel was placed in the same slab tester, preset to −20 °C, and kept at this temperature for 4 h. Afterward, it was removed and kept at ambient temperature for 4 h. The test lasted 40 days (120 cycles). The durability test under extreme conditions (AP-V) involved immersing individual samples in the liquid nitrogen container for 2 min.

**Figure 7:**
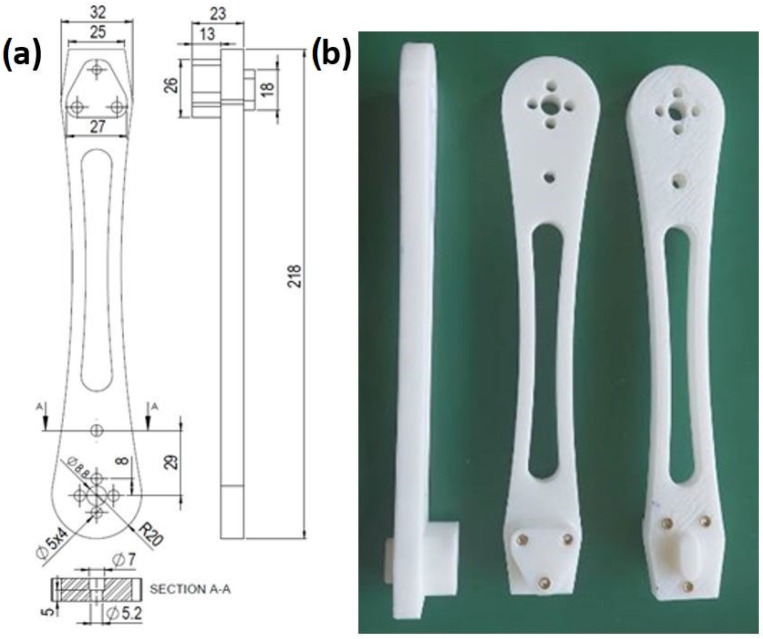
Drone arm: (**a**) schematic view; (**b**) appearance.

Standard “dog bone” samples were 3D printed for each combination of printing parameters. The test was conducted according to EN ISO 572-2 standard. The test speed was 50 mm/min. The Shimadzu compact table-top universal tensile strength tester was used. Three drone arms (dimensions and appearance are given in Figure 7a,b), were fabricated using printing combination IV. The drone arms were kept at ambient temperature for one year (NP-I protocol). Afterward, they were placed in a special tool designed to hold the arm in a cantilevered position. An axial force was continuously applied at the arm’s end until failure. The Instron 1122 universal test device, equipped with a TRC Pro acquisition system and a maximum load capacity of 5 kN, was used. The speed was set to 50 mm/min.

### 2.3. Numerical Analysis—Methodology

In addition to the experimental investigation of the mechanical quality of the 3D printed drone arms, a numerical analysis was conducted to compare its behavior, determine the overall stress state, and assess the onset of material plasticization. The geometric model of the drone arm was designed to fully correspond to the physical model, with a length of 218 mm and a maximum width of 40 mm. The thickness of the drone arm is 10 mm. The input values for elastic modulus, Poisson’s ratio, and specific weight were, respectively, 1605 N/mm^2^, 0.3, and 1240 kg/m^3^. The value of the elastic module was obtained from the tensile strength test results conducted in this study for printing combination IV. A concentrated load of 120 N, acting orthogonally to the mid-plane of the drone arm, was used. This value represents the maximum force that the printed arm withstood during the testing performed in this study. The static analysis of the drone arm was performed using the Finite Element Method (FEM) in the AxisVM software (version 6.50.2.3).

The domain of the drone arm was modeled using a triangular surface finite element mesh. The mesh was generated with an average element side length of 1 mm, with additional refinement applied in the contour regions, where the element side lengths were reduced to less than 1 mm. This triangular finite element comprises six nodes (three at the corners and three at the midpoints of the edges) and is formulated according to Mindlin–Reissner theory, which incorporates the effects of shear deformation. The entire numerical model of the drone arm was treated as a surface model with three degrees of freedom per node: two rotational degrees of freedom about the two orthogonal horizontal axes and one translational degree of freedom in the vertical direction. The model comprises a total of 9423 surface finite elements, with 5103 corner nodes and 14,532 intermediate nodes. A statical analysis was done in AxisVM software. The model had 58,905 equilibrium equations. The entire numerical model of the drone arm was treated as a surface model with three degrees of freedom per node: two rotational degrees of freedom about the two orthogonal horizontal axes and one translational degree of freedom in the vertical direction. The model comprises a total of 9423 surface finite elements, with 5103 corner nodes and 14,532 intermediate nodes. A statical analysis was done in AxisVM software. The model had 58,905 equilibrium equations.

The literature [51,52,53] indicates that the material strength limit, primarily for structural materials (such as drone arms), is obtained from tensile test results. Two distinct domains were analyzed: a linear-elastic domain, where stress and strain increase proportionally, and a nonlinear domain, characterized by material yielding accompanied by significant strain without further stress increase. The boundary between these domains represents the material’s yield point, which was used as a key parameter in the numerical analysis. Specifically, a linear-elastic material model was adopted, with incremental tracking of plasticity initiation through three approaches: principal stresses in two orthogonal directions, maximum principal stresses, and von Mises stress. The normal stress s_yy_ was calculated using Formula (1), where t is the thickness of the drone’s arm and my is the bending moment about the y-axis.

(1) $$s_{yy}=\pm \frac{6}{t^{2}}m_{y}$$

Von Mises stress was computed using Formula (2), where s_xx_,s_yy_,s_zz_ are the normal stresses in the three orthogonal directions, and s_xy_, s_yz_, s_zx_ are the shear stresses.

(2) $$s_{VM}=\sqrt{0.5\left[{\left(s_{xx}-s_{yy}\right)}^{2}+{\left(s_{yy}-s_{zz}\right)}^{2}+{\left(s_{zz}-s_{xx}\right)}^{2}\right]+3\left(s_{xy}^{2}+s_{yz}^{2}+s_{zx}^{2}\right)}$$

The aging effect was incorporated by considering the sample with the most degraded behavior, using its experimentally determined elastic modulus and yield stress.

## 3. Results and Discussion

**Figure 8:**
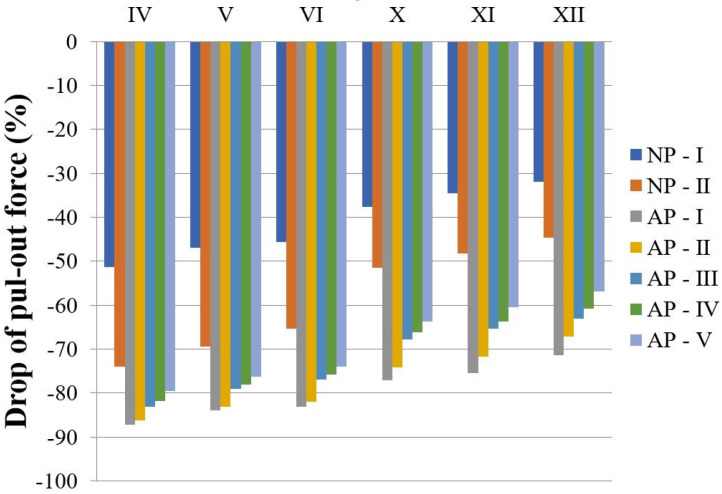
Decrease in pull-out forces detected in conditioned samples in regard to the non-treated SP sample.

Three nominally identical pull-out tests were conducted for each batch. The averaged pull-out values (forces) and the corresponding standard deviations (SDs) obtained from the 3D-printed samples are summarized in Table 3. Figure 8 illustrates the decrease in pull-out forces observed after both natural and artificial aging compared to the initial measurements taken before the aging test, i.e., the SP sample. Both natural and artificial aging significantly influenced the decrease in the pull-out forces measured on all experimental samples. The highest decrease is observed in the case of AP-I, which was expected given that this aging protocol comprised temperature cycling, humidity exposure, dry/wet cycle circumstances, combined load, and environmental exposure to UV and IR radiation. A similar decrease in force is observed with AP-II.

Results in Table 3 suggest that the degradation of the investigated samples caused by freeze–thaw protocols under severe conditions, namely, AP-III and AP-IV, are comparable. Additionally, the extreme freeze technique resulted in a higher pull-out force than those obtained during application of the severe freeze–thaw methods. However, the “enhancing” effect on mechanical properties after extreme exposure was not unexpected, as it was also noted in a previous study that involved PETG samples [54].

**Figure 9:**
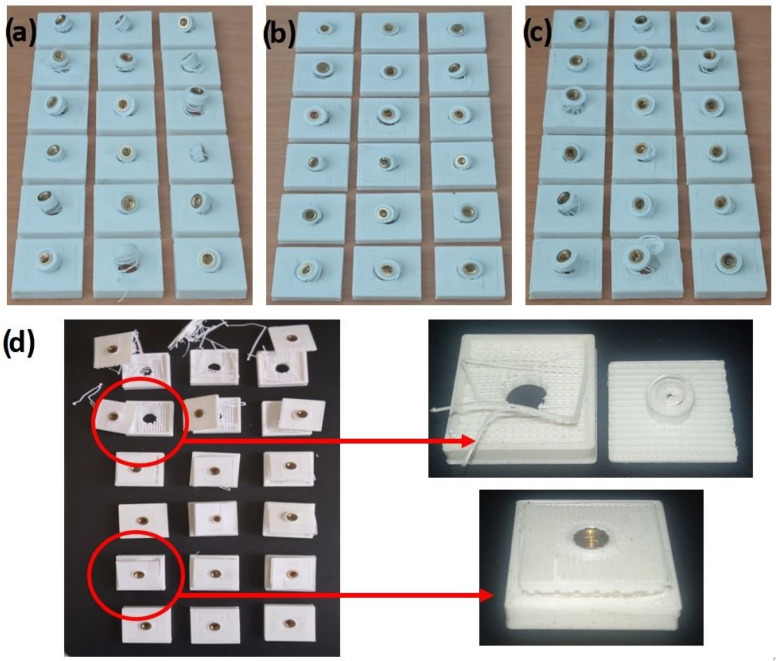
The morphology of the tested samples after the pull-out test: (**a**) NP-II, (**b**) AP-III, (**c**) AP-V, and (**d**) AP-IV.

The morphology of the tested samples after the pull-out test for NP-II, AP-III, AP-IV, and AP-V is illustrated in Figure 9. The samples obtained by natural aging NP-II protocol are illustrated in Figure 9a, while the samples obtained after artificial aging protocols AP-III, AP-V, and AP-IV are given in Figure 9b, 9c, and 9d, respectively. Samples aged under protocols NP-I, AP-I, and AP-II exhibited similar appearance. AP-IV was the only set where a distinct morphology pattern was observed.

Furthermore, each pull-out metal insert was also wrapped in plastic. The thickness of the plastic layer around the implant increased from samples IV to VI and from samples X to XII. This is valid proof that the heat staking insertion method was successful. Since other printing parameters, such as infill density and infill line direction, were constant in the study, it is obvious that the number of wall line contours is responsible for the observed pull-out-force trend, which is consist with the findings reported in the literature [55].

**Figure 10:**
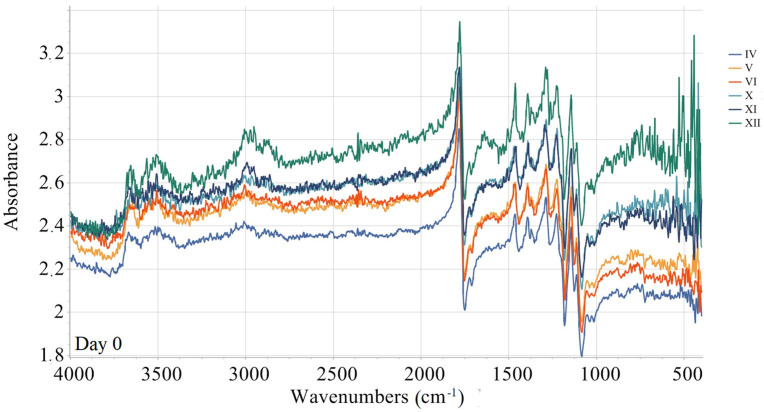
FTIR spectra for samples IV, V, VI, X, XI, XII, AP-I protocol (day 0).

**Figure 11:**
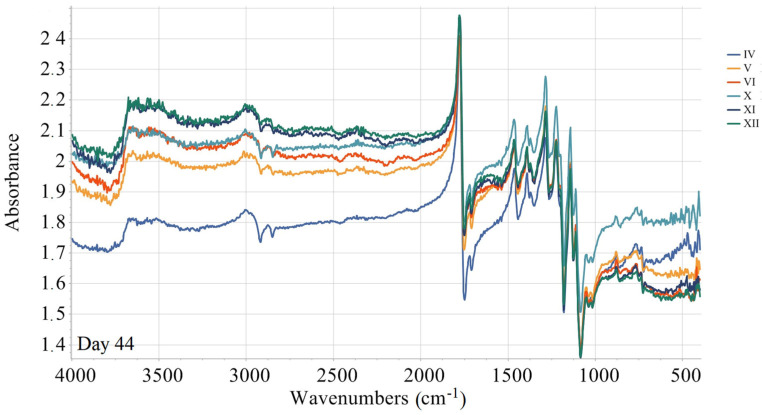
FTIR spectra for samples IV, V, VI, X, XI, XII, AP-I protocol (day 44).

**Figure 12:**
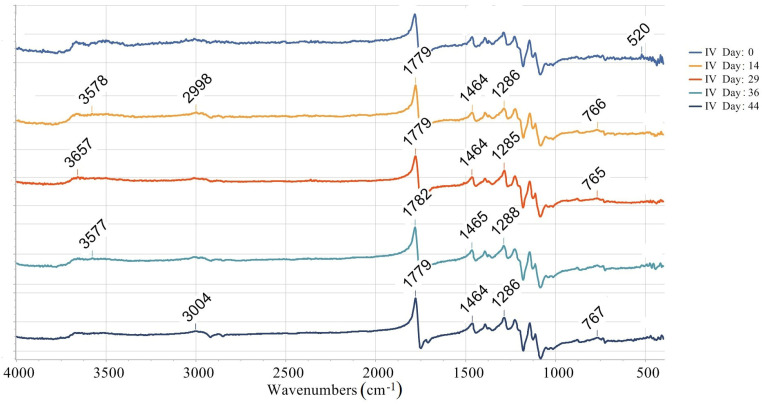
FTIR spectra for the sample IV after 0, 14, 29, 36, and 44 days of aging.

**Figure 13:**
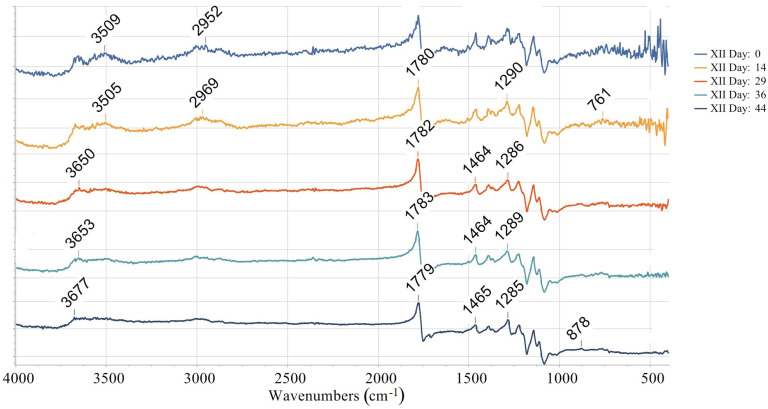
FTIR spectra for the sample XII after 0, 14, 29, 36, and 44 days of aging.

The quantification of the long-term degradation caused by aging is provided for the samples aged under AP-I protocol. FTIR measurements for the mentioned samples are summarized in Figure 10, Figure 11, Figure 12 and Figure 13. Namely, FTIR spectra for AP-I (samples IV, V, VI, X, XI, XII) obtained before aging (day 0) are given in Figure 10, while FTIR spectra for AP-I (samples IV, V, VI, X, XI, XII) obtained after 44 days of aging are provided in Figure 11. FTIR spectra for the samples IV and XII, after 0, 14, 29, 36, and 44 days of aging, are shown in Figure 12 and Figure 13, respectively. Degradation of the samples during 44 days of conditioning was correlated with changes in absorption trends and peak shifts. The absorbance values across the different stages during conditioning (14, 29, 36, and 44 days) showed predominantly consistent behavior, with isolated peaks (changes in intensity) and bands shifting (changes in wavenumbers) over time, which refers to minimal changes in chemical bonds present in the investigated samples.

The absorbance spectrum obtained on the first day of the investigation presents a characteristic baseline with peaks, indicating the initial state of the investigated material (Figure 12 and Figure 13). Minor changes are observed after 14 days of aging, suggesting an initial phase of degradation or even stabilization. After 29 days, the over-all spectrum appears more pronounced in the majority of absorbance peaks, indicating further degradation or alterations. Several peaks emerge, suggesting changes in chemical bonds. After 36 days, the absorbance peaks/bands continue to increase and shift, referring to ongoing degradation. The peaks that show significant intensity changes indicate the possibility of the compound breaking down. At 44 days, the spectral features are mostly pronounced, showing the final extent of degradation (Figure 12 and Figure 13).

The broad peak (3984–3850 nm range) present on the first day is absent at later stages (Figure 12), which can be related to moisture or hydroxyl groups (-OH) disappearing over time. Initial high absorbance (2.85) at wave lines (~1779–1783 nm) that are associated with the stretching vibrations of carbonyl groups (C=O) later slightly decreases (2.40). This suggests slow degradation or oxidation effects on ester bonds. The peaks corelated with the C-H stretch (~3009–3010 nm) remains relatively stable, but its intensity slightly decreases, which indicates minor structural rearrangements but not complete polymer backbone breakdown. Noticeable absorbance fluctuations are registered at wavenumbers connected with C-H bending vibrations and C-C stretch (1464–1465 nm, 1285–1288 nm), referring to the chain scission or fragmentation due to the degradation, as well as rearrangement of the polymer structure. In the region of minor peaks (765–768 nm and 415–416 nm), the absorbance initially decreases after 29 days (to 1.63), followed by an increase after 36 days (to 1.85). This behavior may be attributed to changes between crystalline and amorphous regions.

Overall, the evolution of the absorbance spectra from the 1st to 44th day illustrates a trend of increasing degradation and structural changes. Hydrolytic degradation, oxidating effects, and crystallinity changes are possible FTIR data interpretations. Namely, hydrolytic degradation of PLA occurs via ester hydrolysis, which is evidenced by the reduction in ester peak intensities observed around 1779 nm. The observed changes in the C=O and C–O peaks indicate mild oxidation, which may result from exposure to air. Variations in the peak intensities within the range of 765–768 nm suggest alterations in the material’s amorphous and crystalline structure [25,56,57,58]. Figure 12 and Figure 13 show that the value of absorbance reported at the same wave line increases from sample IV to sample X in each data set. The observed pattern is related with the set printing parameters. Since other printing parameters such as infill density, infill line direction, etc., were constant in the study, this means that the absorbance value is directly correlated with the used number of wall contour lines and its thickness.

**Figure 14:**
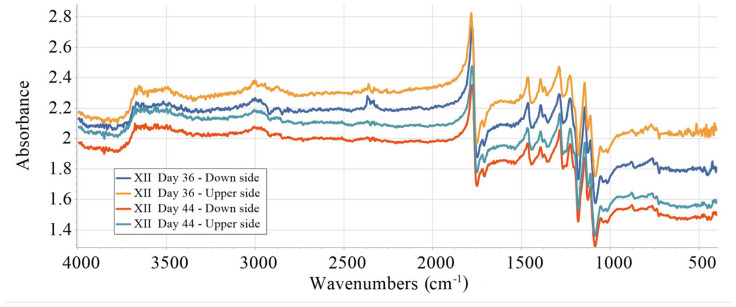
FTIR spectra recorded on the upper and down side of sample XII on 36th and 44th day of aging.

**Figure 15:**
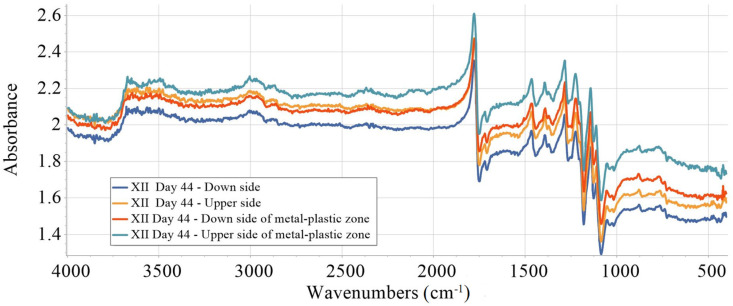
FTIR spectra recorded on the upper and down side of sample XII, both inside and outside the metal–plastic zone, on the 44th day of aging.

The investigated samples were isolated on all sides except the upper side, which remained exposed during the aging process. To assess the extent of deterioration of the samples, FTIR measurements were performed on both sides of the specimens on the 36th and 44th day of aging. In addition, supplementary measurements were conducted in the metal–plastic interface zone on both sides of the samples investigated on the 44th day of ageing. FTIR spectra for the sample XII are given in Figure 14 and Figure 15. The same pattern was observed for all other investigated samples.

A clear and progressively more pronounced decrease in absorbance over time, especially on the exposed (upper) surface, is evident in Figure 14 across key FTIR regions (e.g., 3600, 2900, 2300, 1750, 1200, and 750 cm^−1^). In comparison, the unexposed (down) surface exhibits relatively minor changes, which is consistent with its protection from direct exposure. For a more detailed assessment, the degradation index was calculated. The integration of peak areas in the selected FTIR regions was performed using Spectragryph v.1.2.16.1 software. The corresponding results are presented in Table 4, and the degradation index was computed using Formula (3).

(3) $$DI=\frac{{Inegral\;}_{Day\;36}-{Inegral\;}_{Day\;44}}{{Inegral\;}_{Day\;36}}\times 100\%$$

The O–H stretching region (3757–3485 cm^−1^) showed an increase in intensity on both sides, especially on the down side (−54.6% DI), likely reflecting the formation of hydroxyl groups resulting from hydrolytic degradation. The C–H stretching region (2836–3054 cm^−1^), associated with the polymer backbone, also showed a considerable increase on the down side (−48.0% DI), while a moderate reduction was observed on the upper side (15.9%), indicating possible chain scission. A significant reduction in the C=O shift region (2439–2255 cm^−1^) was observed on the down side (61.4% DI), in contrast to a slight increase on the upper side (−7.6% DI), suggesting different degradation mechanisms. The main carbonyl ester region (1893–1649 cm^−1^) displayed an unusual trend, with a decrease on the down side (13.7% DI) but a significant increase on the upper side (−131.7% DI), pointing to the formation of new ester or acid groups.

In the fingerprint region (1536–989 cm^−1^), corresponding to C–O, C–C, and skeletal vibrations, both sides showed moderate changes (13.9% on the down side, 3.3% on the upper side). Finally, the crystallinity-sensitive region (805–693 cm^−1^) increased significantly on the upper side (−150.5% DI), possibly indicating recrystallization or chain ordering induced by degradation, while changes on the down side were minimal (6.2%).

The unexposed (down) sample side demonstrated more consistent degradation across the C=O, backbone, and skeletal regions, whereas the upper side showed signs of reorganization or surface oxidation, particularly in the ester and crystallinity regions. Overall, the down side of the sample exhibited clear signs of degradation, especially in the carbonyl and skeletal bands, while the upper side experienced less degradation and possibly some reorganization or oxidation-related changes. These findings point to an inhomogeneous aging process across the sample’s thickness.

The integration of peak areas in the selected FTIR regions was performed initially. The obtained results were used to compute the degradation index (DI%) relative to the down side value after 44 days of aging. The results are summarized in Table 5.

The given spectra changes are relatively more stable, with only minor signs of surface reorganization. The analysis reveals distinct degradation patterns across different regions. The exposed (upper) PLA side relative to the unexposed (down) PLA side exhibits only slight increases in all regions, with negative DI values ranging from approximately −4% to −8%, indicating minimal changes and suggesting that surface exposure differences or localized interactions have little impact on overall degradation. Conversely, the down side, particularly within the metal–plastic zone, shows moderate to significant increases in key regions; for example, the C–H stretch exhibits a decrease of around −36.98%, and the carbonyl (C=O) region increases by about −15.12%. These changes point to enhanced chain scission and hydroxyl formation, characteristic of advanced hydrolysis near the interface. The accumulation of degradation products such as esters or acids is supported by the increases observed in the main carbonyl and skeletal vibration regions. Interestingly, the slight decrease in the CO_2_/ester region (positive DI of 7.85%) may reflect partial volatilization or transformation of low molecular weight species. The upper side in the metal–plastic zone displays the most pronounced degradation, with a significant decrease, up to approximately −71.8% in the C–H region and −70.5% in the CO_2_/ester region, as well as substantial rises in O–H and main carbonyl regions. These findings indicate significant backbone cleavage, which was probably intensified by thermal effects at the metal–plastic interface due to heat accumulation in the brass metal. Despite these chemical changes, the skeletal region remains relatively stable, with only about a 1.3% increase, indicating partial retention of the polymer’s main structure. A slight increase in crystallinity (~5.5%) may be attributed to chain reordering or recrystallization of degraded segments. The metal–plastic interfaces, particularly on the upper surface, show significantly higher degradation levels, likely due to the combined effects of heat, sunlight, and moisture. In contrast, out of the metal–plastic zone, the upper and down sides show only mild changes, pointing to more uniform but limited aging processes. The key indicators of degradation include increases in the O–H and carbonyl regions, consistent with hydrolytic and oxidative mechanisms that are typical for PLA. The results clearly suggest that degradation is more pronounced on the down surface and at the metal–plastic interface, particularly in the hydrocarbon and carbonyl regions, indicating non-uniform aging patterns.

**Figure 16:**
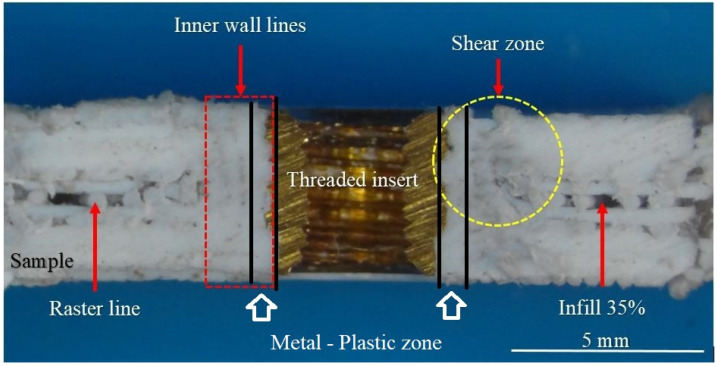
The cross section of Sample XII (AP-V) after the pull-out test.

The cross section of the sample XII, which was initially aged under extreme conditions AP-V protocol and afterwards tested using the pull-off test, is reported in Figure 16 as an example. This sample can also be seen in Figure 9 as the third specimen in the last row. It is evident that the adhesion between the metal and plastic is kept after the pull-out test, and that the material has lost its load bearing capacity in the shear zone. Actually, the weakest point is registered just at the end of the metal–plastic zone. This zone was formed during the heat staking process.

Material properties in this zone are different than the properties out of it. On the 44th day, the comparison of FTIR absorbance values taken from Figure 15 indicates notable chemical differences between the exposed surfaces and the metal–plastic zone. The hydroxyl stretching region (3757–3485 cm^−1^) exhibited the highest absorbance in the metal–plastic zone, particularly on the upper side, suggesting an increased presence of hydroxyl groups, likely associated with hydrolytic degradation and moisture uptake. Similarly, higher absorbance in the C–H stretching region (2836–3054 cm^−1^) in the metal–plastic zone may reflect an accumulation of backbone fragments or partially cleaved polymer chains. Elevated values observed in the carbonyl-related regions (2439–2255 cm^−1^ and 1893–1649 cm^−1^), especially in the upper metal–plastic zone, imply the formation of degradation products such as esters or volatile acids, or other oxidation byproducts. These findings suggest that chemical changes are more advanced in the metal—plastic zone, possibly due to localized thermal effects, the presence of metal ions, or moisture retention. The skeletal region (1536–989 cm^−1^) showed moderate variation, with slightly higher values in the metal–plastic zone, potentially indicating the presence of dense skeletal structure or accumulation of degradation fragments. Furthermore, increased absorbance in the crystallinity-associated region (805–693 cm^−1^) may point to partial recrystallization or increased molecular ordering in partially degraded segments.

**Figure 17:**
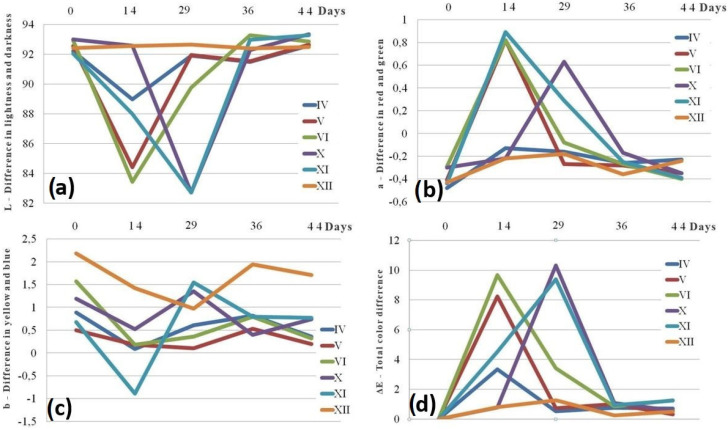
Colorimetric analysis: (**a**) change of L during aging; (**b**) change of a during aging; (**c**) change of b during aging; and (**d**) color difference ∆E.

The results of the colorimetric analysis are given in Figure 17a–d. The sample XII maintained a highly stable difference in lightness and darkness—L value—throughout the aging process (Figure 17a). As aging progressed, a significant decrease in L values was observed after 14 days for samples IV to VI, and on the 29th day for samples X and XI. After the initial decrease, a stabilization phase was noted. The difference in red and green—a value—remained relatively stable for samples IV and XII during the entire period (Figure 17b). For the remaining samples, this value exhibited a pattern similar to that of L, with an initial drop followed by stabilization. The difference in yellow and blue—b value—showed the maximal decrease after 14 days for samples IV, VI, X, and XI, after which the value rose, and after 29 days it was above the baseline, indicating that the stabilization phase has begun (Figure 17c). For samples V and XII, the **b** values reached their lowest point at the 29th day and began to stabilize from the 36th day. The total color difference—ΔE value—is visualized in Figure 17d. The ΔE values exceeding 2 are typically considered noticeable to the human eye. In this context, significant color changes were observed for samples V, VI, X, and XI, particularly after 14 and 29 days. These changes were later followed by a period of stabilization or partial recovery. The sample XII exhibited the most consistent color stability over time. These findings are in agreement with the results obtained from pull-out force testing and FTIR analysis.

**Figure 18:**
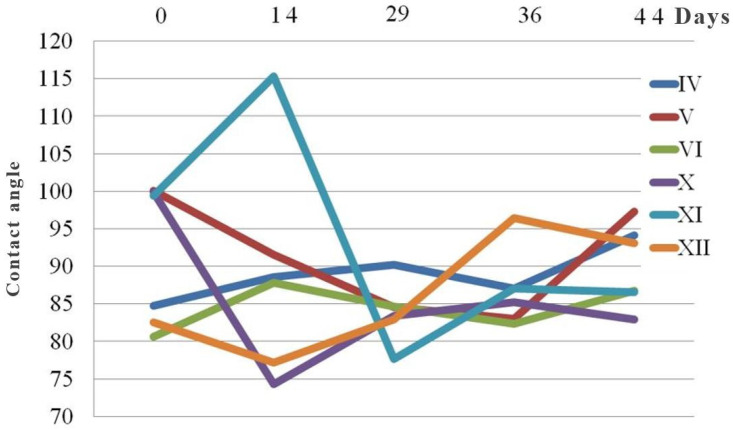
Change of contact angle during aging.

The contact angle measurements are summarized in Figure 18. The resulting data indicate varying degrees of stability and hydrophobicity among the different PLA samples over the designated aging period. While certain samples, such as IV and VI, demonstrate consistent improvement, others display fluctuations in surface properties, likely due to environmental sensitivity or degradation processes.

Specifically, the hydrophobicity of the sample IV improves with age due to degradation, which causes hydrophobic groups to become increasingly exposed on the surface. In sample V, the contact angle is maximum at the beginning. After 36 days, the value dropped to 83.02. From this point on, its value is rose until it reached a level near to the baseline. A relatively stable performance with approximately gradual improvement in hydrophobicity was observed for sample VI. A significant loss in hydrophobicity followed with a slight recovery was observed for sample X. An unusual and unexpected high contact angle of 115° was registered for sample XI after 14 days. After that, a decreasing trend was registered before the material reached stability. In the case of sample XII, an improvement in hydrophobicity after certain initial fluctuations was also observed.

As can be seen from FTIR data interpretation (Figure 10, Figure 11, Figure 12 and Figure 13), PLA degradation follows three stages: hydrolysis, oligomer formation, and crystallinity increase. During hydrolysis, ester bonds are cleaved, which will led to the increased hydrophilicity. During oligomer formation, the chains become smaller. This occurrence inevitably led to the variable surface properties. As crystallinity increases, degraded amorphous regions become more hydrophobic, which means that in cases of samples where the contact angle indicates a shift towards more hydrophobic surface, crystallinity or surface roughening is the probable degradation mechanism, such as in experiment IV (steady increase) and experiment VI (upward trend after an initial drop). In the samples where the contact angle is causing a shift towards more hydrophobic surfaces, hydrolysis is the dominating degradation mechanism. Representative examples are samples X (a sharp drop) and XI (a falling trend after its peak). When there is a large variability in the contact angle, the substance will simultaneously undergo hydrolysis and crystallization. Experiment V (a dip and recovery) is a typical example of such behavior.

**Figure 19:**
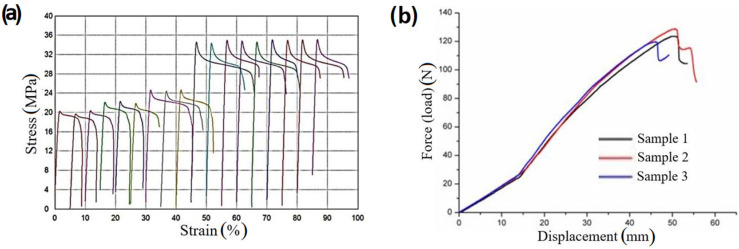
Mechanical testing of the 3D-printed drone arms: (**a**) tensile strength test; (**b**) concentration load test.

The results of the mechanical testing of the 3D-printed drone arms, i.e., the tensile strength and concentrated load tests after one year of natural aging, respectively, are given in Table 6 and Figure 19. The modulus of elasticity increases significantly from samples VI to XII. A higher modulus suggests that these samples can withstand greater stresses while maintaining a high degree of stiffness, which is beneficial for drone applications.

A clear increasing trend in maximum stress and strain from groups IV to XII is visible in Figure 19. Samples X, XI, and XII have significantly higher stress than those in groups IV, V, and VI. The average maximum stress in the cases of samples IV and V is between 18 and 19 MPa. The corresponding strain values for these samples remains relatively low, indicating a brittle behavior.

**Figure 20:**
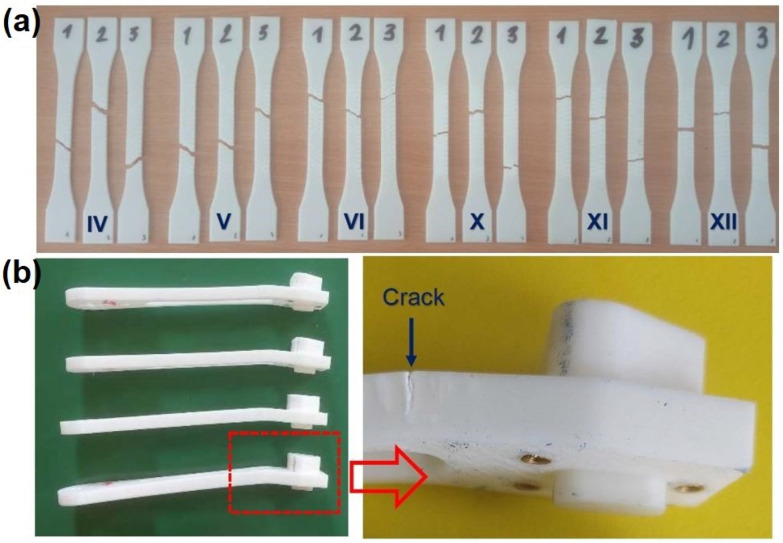
The sample morphology after: (**a**) tensile strength testing; (**b**) concentration load testing.

Sample VI has a slight increase in average maximum stress (i.e., tensile strength) in comparison to samples IV and V. Furthermore, greater variability in strain levels is found, which leads to a moderate strength and ductility improvement of this sample in comparison to previous ones. Maximum stress (i.e., tensile strength) for samples X–XII is around 28 MPa. The substantial elongation, especially in samples X-1 and XI-2, was registered, which is a clear indication that samples X–XII are more ductile. The morphology of samples after tensile strength tests, which is shown in Figure 20, is in line with the observed ductility pattern. Samples IV and V are mostly brittle, while samples VI–XII are more ductile.

Figure 19b shows that the force–displacement relationship during the concentration load test is initially linear, indicating the elastic behavior of the drone arm up to a load of approximately 25 N. Beyond this point, the displacement increases faster, indicating a change in slope and the onset of plastic deformation or yielding. Near the maximum applied force, with an average value of 127 N, a significant reduction in stiffness is observed, marking the transition to material failure.

**Figure 21:**
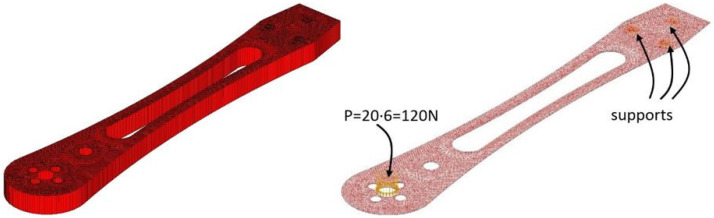
Isometric view of the numerical model of the drone arm.

Figure 21 shows the drone arm with applied self-weight (uniformly distributed over the surface), along with the supports and place where concentrated force was applied. A concentrated force of 120 N, acting orthogonally to the mid-plane of the drone arm, was used. This value represents the maximum force that the printed arm withstood during concentration load testing. Since this force is exerted at the central hole of the arm, it was redistributed along the hole’s perimeter into 20 equal segments. Additionally, the supports were modeled as annular line supports with stiffness components in all three orthogonal directions (kx = ky = kz = 10^10^ kN/m/m), while the rotational stiffness was set to zero.

**Figure 22:**
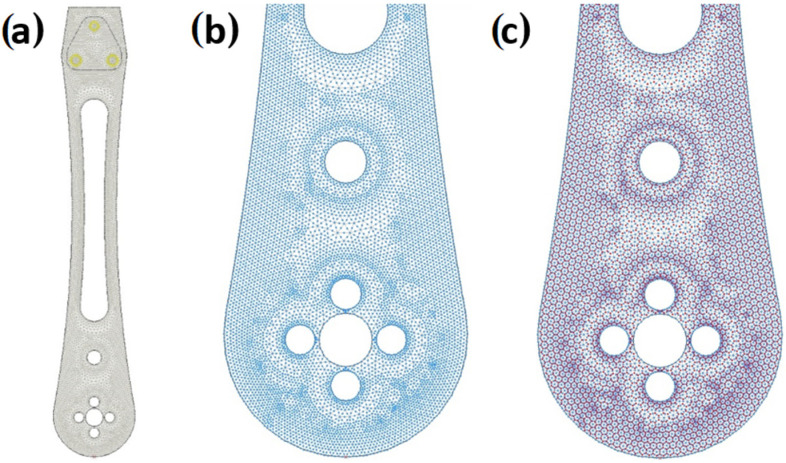
Generated finite element mesh: (**a**) complete model; (**b**) detail of the mesh with corner nodes; and (**c**) detail of the mesh with corner and central nodes.

The generated finite element mesh for the complete model (described in Section 2.3) is given in Figure 22 (complete model is provided in Figure 19a. A detail of the mesh with corner nodes and a detail of the mesh with corner and central nodes are given in Figure 19b). Forces in the cross-section of the drone arm include the moments m_x_, m_y_, m_xy_, and shear forces q_x_, q_y_. The global axes are the horizontal X- and vertical Y-axis. The main influences are defined as m_1_, m_2_, angle α_n_, and the resultant shear force q_R_. The main directions of influence were direction 1 and direction 2.

**Figure 23:**
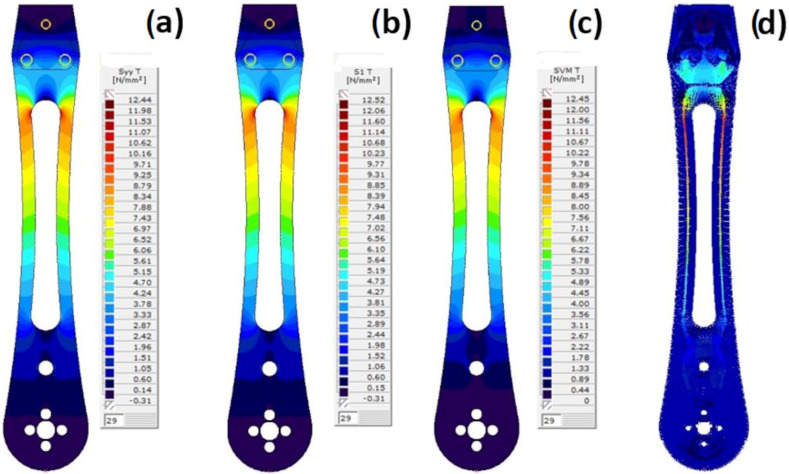
The stress state iso-surface (top surface of the drone arm) for a force increment of 0.1·P: (**a**) s_yy_; (**b**) s_1_; (**c**) s_VM;_ and (**d**) s_1_ and s_2_ trajectories.

The evolution of stress states and their distribution in the drone arm within the linear behavior domain was calculated using the force increment of 0.1. The change of the stress disposition is illustrated only for the upper surface of the drone arm, since all dominant load-bearing stresses are occurring on this side. The stress state iso-surfaces are shown for stresses s_yy_ in the Y-axis direction, stresses s_1_ in the principal direction 1, and von Mises stresses s_VM_ in Figure 23.

The results presented in Figure 23 indicate that all three stress states have similar yield values. However, to better identify and analyze the region of material plasticity development and crack initiation, a further investigation was carried out based on von Mises stresses, which incorporated the effects of s_xx_, s_yy_, and s_xy_ stresses. The appearance of cracking is attributed to the exceedance of the material’s tensile yield strength under bending, caused by the aforementioned perpendicular loading. At the critical cross-section where plastic deformation initiates, both tensile and compressive stresses are simultaneously present. This stress state arises from the action of the bending moment, which results in the elongation of one edge of the section due to tension and the contraction of the opposite edge due to compression.

The yield strength limit of the material was taken from uniaxial tensile strength tests. Accordingly, the onset of plastic deformation in the drone arm is considered the serviceability threshold, as the arm retains a certain level of structural integrity beyond this point. The functional performance of the component is compromised once cracking initiates in the cross-section, rendering the arm no longer operational. Thus, functional failure corresponds to the serviceability limit state. An example of the analytical calculation is given in Table 7 for sample IV-3.

Numerical analyses of the drone arm were carried out using an iterative procedure, starting with an initial scaling factor of 0.15 for the concentrated force set at 120 N. This value was then progressively increased in increments of 0.01.

**Figure 24:**
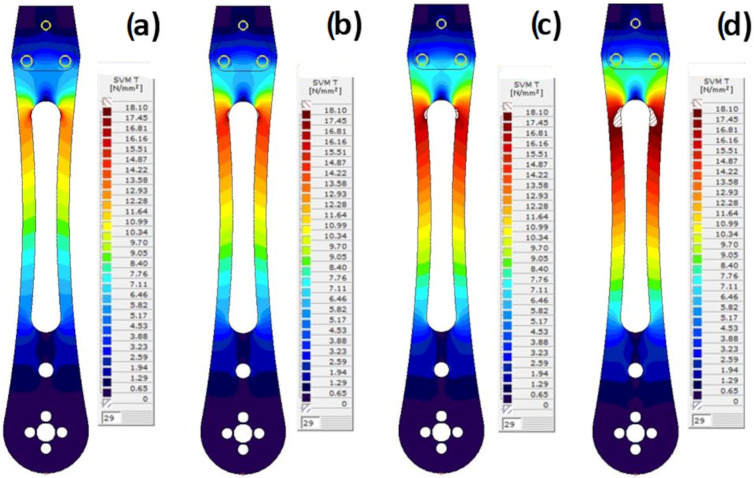
Von Mises stress state iso-surface (arm top surface) for various force increments (i.e., increment factor): (**a**) 0.15; (**b**) 0.17; (**c**) 0.19; and (**d**) 0.21.

The von Mises iso-surfaces stress states for the scaling factors 0.15, 0.17, 0.19, and 0.21 are given in Figure 24. The zone of plasticity initiation is now clearly identified. This process takes place from the inner edge to the outer edge of the drone arm.

**Figure 25:**
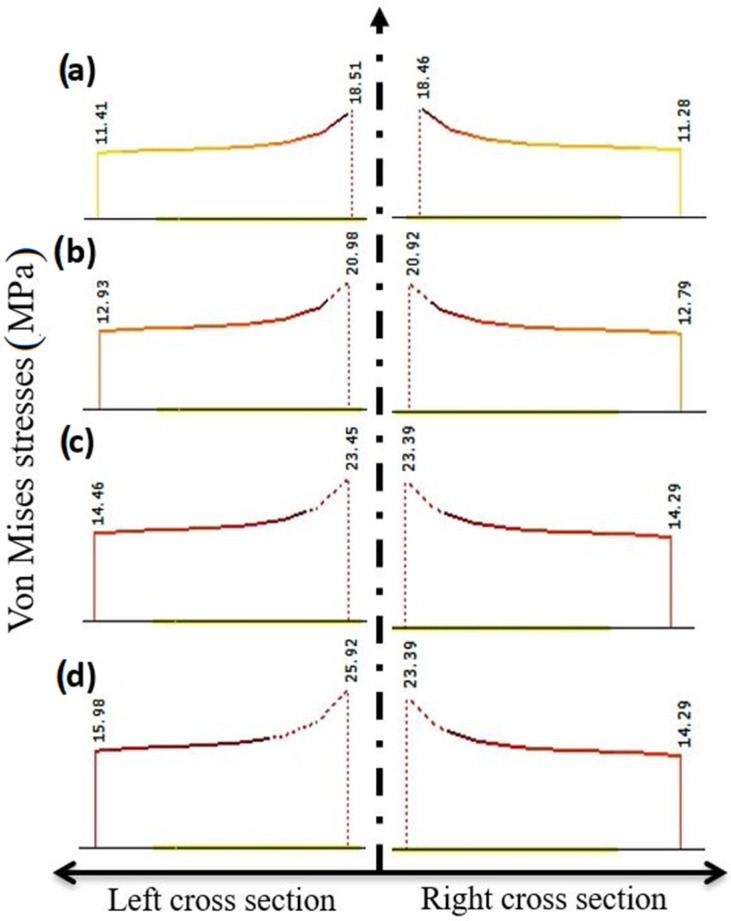
Von Mises stresses in the section where yielding occurs for various scaling factors (i.e., increment factor): (**a**) 0.15; (**b**) 0.17; (**c**) 0.19; and (**d**) 0.21.

The development of von Mises stresses in the cross-section where yielding occurs is given at Figure 25 for the same scaling factors. It is obvious that stresses are not evenly distributed across the cross-section; indeed, they are significantly higher at the inner side of the drone’s arm. This means that yielding initially develops from the inner side and progresses to the outer side of the drone’s arm.

The calculated average allowed concentration loads (ACLs) that the drone arm can hold without entering into the plastic zone with confidence levels of 10% and 20% are summarized in Table 8 for all samples.

It is important to note that this is excellent output, since the infill density and raster line in this study were set to 35% and 0.3, respectively. It is generally understood that the mechanical properties of printed parts are directly related to the infill density and raster line. The common value of infill density for high quality parts is around 70%. It was proven that PLA filament is suitable for drone application. Additionally, it was observed that even the aged drone arms printed with the low infill density can withstand a high payload capacity (1.46–2.31 kg).

## 4. Conclusions

This study demonstrated that PLA–brass threaded joints, inserted by the heat staking procedure, maintained their mechanical functionality following natural and artificial aging, confirming their reliability and suitability for use in the drone industry. The one group of samples aged under the most aggressive aging protocol (AP-I) notably retained a pull-out force of 17.9 kg. Moreover, artificial aging protocols were developed and applied, incorporating thermal cycling, humidity, UV and IR radiation, and freeze–thaw conditions. This is the first reported use of such a comprehensive aging protocol for evaluating PLA–brass joints, setting a new experimental standard for aging studies in hybrid 3D-printed materials.

Despite a relatively low infill density of 35%, the 3D-printed drone arms exhibited substantial load-bearing capacity. After one year of natural aging, each arm could support up to 2.31 kg. These findings challenge the conventional assumption that high infill densities are essential for structural reliability in additive manufacturing. When raster line spacing and infill density were held constant, the number of wall contour lines and thickness emerged as the most influential parameters affecting mechanical strength, degradation resistance, and joint performance.

FTIR spectroscopy, supported by degradation index (DI) calculations and absorbance trends, provided clear evidence of chemical changes in PLA during aging. The results show that environmental exposure, particularly in artificial aging protocols, increases hydroxyl and carbonyl group intensities, indicating the polymer matrix’s progressive hydrolytic and oxidative degradation. The absorbance values across key FTIR bands (e.g., O–H, C=O, and C–H) increased significantly over time, especially on the exposed surfaces and within the metal–plastic interface zone. These regions exhibited the highest DI values, confirming that aging most severely affects the area surrounding the embedded brass inserts. The data further revealed that degradation was not uniform: samples’ down (unexposed) sides showed less chemical change than the upper (exposed) sides, and the metal–plastic contact zones exhibited the most intense degradation signals. This localized deterioration aligns with the observed mechanical weakening in pull-out tests. The identification of spatially uneven degradation (top vs. bottom surfaces, metal interface vs. bulk) is particularly novel and impactful for the design of more durable polymer–metal assemblies.

The cross-section of the group of samples initially aged under extreme conditions according to the AP-V protocol and subsequently tested using a pull-out test was also analyzed. It was found that the adhesion between the metal and plastic remains intact after the pull-out test, although the material had lost its load-bearing capacity in the shear zone. The weakest point is observed at the end of the metal–plastic interface, a zone formed during heat staking.

Numerical simulations using the Finite Element Method (FEM) supported the experimental findings, showing that material yielding initiates at the inner edge of the drone arm and progresses outward. This is in line with the observed load paths and deformation behavior. A custom adapter was also designed to ensure consistent and repeatable pull-out testing.

Future research will evaluate the mechanical performance of arms printed with higher infill densities (70–90%) and varied raster lines to optimize strength and durability further. Testing higher densities can optimize strength-to-weight ratios for specific load cases in drone design. Finally, sustainability aspects such as material efficiency and life cycle impacts warrant further study, especially in light of the promising mechanical performance observed in low-infill structures.

## Figures and Tables

**Table 1:** Creality [41] CR-PLA filament characteristics.

Property	Range/Value
Density	1.25 ± 0.05 g/cm^3^
Water absorption	0.5%
Extruder temperature	190–230 °C
Bed temperature	max. 60 °C
Printing speed	40–100 mm/s
Tensile strength	61 MPa
Bending strength	69 MPa
Flexural strength	78 MPa
Impact strength	0.0105 KJ/m^2^

**Table 2:** Printing parameter combinations.

Sample Designation Code	IV	V	VI	X	XI	XII
Wall thickness (mm)	0.8	1.2	1.6	0.8	1.2	1.6
Wall line contour	2	3	4	2	3	4
Top layer number	2	2	2	4	4	4
Bottom layer number	2	2	2	4	4	4

**Table 3:** The average pull-out forces obtained from the samples after natural and artificial aging.

Designation Code	Pull-Out Force (N)							
	SP	NP-I	NP-II	AP-I	AP-II	AP-III	AP-IV	AP-V
IV	433	211	113	55	60	73	79	89
(SD)	0.50	1.95	1.85	1.73	1.90	1.65	1.77	1.60
V	442	235	135	71	75	93	97	105
(SD)	0.40	1.20	1.50	1.20	4.90	4.10	4.70	3.10
VI	505	275	175	85	91	116	122	131
(SD)	0.26	1.32	1.12	1.08	1.30	1.22	1.12	1.70
X	578	360	280	132	149	186	195	210
(SD)	0.50	1.30	1.02	1.15	1.74	1.95	1.02	1.95
XI	619	405	320	152	175	215	225	245
(SD)	0.35	1.70	1.15	1.30	1.50	1.25	1.90	1.70
XII	624	425	345	179	205	230	245	269
(SD)	0.85	1.90	1.15	1.75	1.57	1.45	1.95	1.30

**Table 4:** Degradation index.

FTIR Region (cm^−1^)	Region Type	Sample Side	FTIR Region Integral Value		Degradation Index
			Day 36	Day 44	DI (%)
3757–3485	O–H stretch (hydroxyl)	Down side	8.354	12.91	−54.6% (↑ OH)
		Upper side	13.02	13.66	−4.9% (↑ OH)
2836–3054	C–H stretch (backbone)	Down side	2.157	3.193	−48.0% (↑ CH)
		Upper side	3.963	3.333	15.9%
2439–2255	C=O shift (CO_2_/ester)	Down side	4.002	1.546	61.4%
		Upper side	1.504	1.618	−7.6% (↑)
1893–1649	C=O ester (main)	Down side	9.834	8.485	13.7%
		Upper side	3.846	8.910	−131.7% (↑)
1536–989	C–O, C–C, skeletal	Down side	54.39	46.82	13.9%
		Upper side	51.16	49.48	3.3%
805–693	Crystallinity/order	Down side	1.290	1.210	6.2%
		Upper side	0.523	1.310	−150.5% (↑)

**Table 5:** Degradation index relative to the down side value obtained at aging day 44.

FTIR Region (cm^−1^)	Region Type	Degradation Index DI (%)		
			Metal–Plastic Zone	
		Upper Side	Down Side	Upper Side
3757–3485	O–H stretch (hydroxyl)	−5.10	−14.39	−17.01
2836–3054	C–H stretch (backbone)	−4.20	−36.98	−71.80
2439–2255	C=O shift (CO_2_/ester)	−4.25	+7.85	−70.51
1893–1649	C=O ester (main)	−4.87	−15.12	−14.78
1536–989	C–O, C–C, skeletal	−5.65	−10.79	+1.36
805–693	Crystallinity/order	−7.93	−12.15	−5.54
FTIR Region (cm^−1^)	FTIR Region Integral Value			
	Out of Metal–Plastic Zone (PLA Zone)		Metal–Plastic Zone	
	Upper Side	Down Side	Upper Side	Down Side
3757–3485	13.00	12.37	14.47	14.15
2836–3054	3.326	3.192	5.483	4.372
2439–2255	1.621	1.555	2.652	1.433
1893–1649	8.902	8.488	9.743	9.770
1536–989	49.46	46.81	46.16	51.87
805–693	1.306	1.210	1.277	1.357

**Table 6:** Results of the mechanical testing of the 3D-printed drone arms.

Sample	Maximal Stress, σ (MPa)	Strain at Maximal Stress, ε (%)	Modulus of Elasticity, E (MPa)
IV-1	18.07	8.22	1056
IV-2	17.70	7.92	1605
IV-3	18.07	8.59	1253
V-1	19.23	9.19	1276
V-2	19.41	8.43	1083
V-3	18.51	9.20	1762
VI-1	20.28	14.48	1416
VI-2	20.22	12.95	1853
VI-3	20.26	11.57	1851
X-1	27.18	20.30	3522
X-2	28.52	11.61	2520
X-3	28.79	12.42	2705
XI-1	28.12	15.64	2962
XI-2	27.78	15.19	2691
XI-3	28.86	9.84	2375
XII-1	28.24	12.44	2901
XII-2	28.04	15.38	2388
XII-3	28.52	11.94	2800

**Table 7:** The analytical calculation example.

Parameter	Symbol/Formula	Value	Unit
Applied force (perpendicular to leg axis)	P	120	N
Moment arm (to critical section)	r	148	mm
Critical section width (2 × 6 mm)	b	12	mm
Critical section height	h	10	mm
Yield stress of plastic	fy	20	MPa
Bending moment	M = P × r	17,760	N·mm
Moment of inertia of the section	I = (b × h^3^)/12	1000	mm^4^
Section modulus	W = I/(h/2)	200	mm^3^
Maximum stress at the critical section	fmax = M/W	88.8	MPa
Yield stress	fy	18.1	MPa
fmax > fy	88.8 > 18.1	Yes	—
Critical force causing plasticity	Pcr = (fy × W)/r	24.46	N
Applied force (P = 120 N) > Pcr	120 > 24.46	Yes (plasticity)	—
Confidence level 10%	CL = fy × 0.90	16.29	N
Confidence level 20%	CL = fy × 0.80	14.48	N
Allowed concentration arm load 10%	ACL = 10/9.81	1.66	kg
Allowed concentration arm load 20%	ACL = 20/9.81	1.47	kg

**Table 8:** Allowed concentration loads for drones.

Sample	IV	V	VI	X	XI	XII
ACL 10% per drone arm (kg)	1.59	1.67	1.78	2.48	2.50	2.70
ACL 20% per drone arm (kg)	1.46	1.53	1.63	2.38	2.29	2.31
Drone payload capacity with ACL 10% (kg)	6.36	6.68	7.12	9.92	10	10.8
Drone payload capacity with ACL 20% (kg)	5.84	6.12	6.52	9.52	9.16	9.24

## Data Availability

The original contributions presented in this study are included in the article. Further inquiries can be directed to the corresponding author.
